# Inflammatory Cells in Adipose Tissue and Skeletal Muscle of Patients with Peripheral Arterial Disease or Chronic Venous Disease: A Prospective, Observational, and Histological Study

**DOI:** 10.3390/jcdd11040121

**Published:** 2024-04-16

**Authors:** Joana Ferreira, Adhemar Longatto-Filho, Julieta Afonso, Susana Roque, Alexandre Lima Carneiro, Isabel Vila, Cristina Silva, Cristina Cunha, Amílcar Mesquita, Jorge Cotter, Margarida Correia-Neves, Armando Mansilha, Pedro Cunha

**Affiliations:** 1 Vascular Surgery Department–Fisiologia e Cirurgia, Centro Hospitalar Universitário de São João, 4200-319 Porto, Portugal; 2 Life and Health Science Research Institute (ICVS), School of Medicine, University of Minho, 4710-057 Braga, Portugaljulietaafonso@med.uminho.pt (J.A.);; 3 Centro Académico Hospital da Senhora da Oliveira, 4835-044 Guimarães, Portugalcristinasmsilva@gmail.com (C.S.);; 4 ICVS/3B’s–PT Government Associated Laboratory, 4710-057 Braga, Portugal; 5 Department of Pathology (LIM-14), University of São Paulo School of Medicine, São Paulo 01246-903, SP, Brazil; 6 Molecular Oncology Research Center, Barretos Cancer Hospital, Barretos 14784-400, SP, Brazil; 7 Radiology Department, ULSAM—Hospital de Santa Luzia, 4904-858 Viana do Castelo, Portugal; 8 Medicine Department, Hospital da Senhora da Oliveira, 4835-044 Guimarães, Portugal; 9 Center for the Research and Treatment of Arterial Hypertension and Cardiovascular Risk, Internal Medicine Department, Hospital da Senhora da Oliveira, 4835-044 Guimarães, Portugal; 10 Vascular Surgery Department, Hospital da Senhora da Oliveira, 4835-044 Guimarães, Portugal

**Keywords:** inflammation, skeletal muscle, adipose tissue, atherosclerosis, peripheral arterial disease, varicose veins, chronic venous disease

## Abstract

The main goal of this study was to assess whether the presence of peripheral arterial disease (PAD) correlates with increased inflammatory cell infiltration. An observational, single-centre, and prospective study was conducted from January 2018 to July 2022. Clinical characteristics and anthropometric measures were registered. Consecutive PAD patients with surgical indications for a common femoral artery approach and patients with varicose veins with an indication for surgical ligation of the saphenofemoral junction were included. In both groups, samples of sartorius skeletal muscle, subcutaneous adipose tissue (SAT), and perivascular adipose tissue (PVAT) were collected from the femoral region. We analysed the characteristics of adipocytes and the presence of haemorrhage and inflammatory cells in the samples of PVAT and SAT via haematoxylin–eosin staining. We found that patients with PAD had significantly more inflammatory cells in PVAT [16 (43.24%) vs. 0 (0%) *p* = 0.008]. Analysing SAT histology, we observed that patients with PAD had significantly more CD45+ leucocytes upon immunohistochemical staining [32 (72.73%) vs. 3 (27.27%) *p* = 0.005]. Upon analysing skeletal muscle histology with haematoxylin–eosin staining, we evaluated skeletal fibre preservation, as well as the presence of trauma, haemorrhage, and inflammatory cells. We registered a significantly higher number of inflammatory cells in patients with PAD [well-preserved skeletal fibres: PAD = 26 (63.41%) vs. varicose veins = 3 (37.50%) *p* = 0.173; trauma: PAD = 4 (9.76%) vs. varicose veins = 2 (25.00%) *p* = 0.229; haemorrhage: PAD = 6 (14.63%) vs. varicose veins = 0 (0%) *p* = 0.248; inflammatory cells: PAD = 18 (43.90%) vs. varicose veins = 0 (0%) *p* = 0.018]. Patients with PAD had a higher number of inflammatory cells in skeletal muscle and adipose tissue (PVAT and SAT) when compared with those with varicose veins, emphasizing the role of inflammation in this group of patients.

## 1. Introduction

Peripheral artery disease (PAD) is characterized by arterial stenosis and occlusions of the arteries in the lower limbs and is one of the most common manifestations of atherosclerosis [1,2,3]. PAD affects more than 202 million people worldwide, with more than 20% of those aged over 65 years old suffering from this condition [2,4,5,6]. It represents the third most common localization of atherosclerosis, after coronary heart disease and cerebrovascular disease [7]. Atherosclerosis is a progressive inflammatory disease in which lipids, extracellular matrix elements, and activated vascular smooth muscle cells accumulate in the arterial wall, resulting in the growth of an atherosclerotic plaque [8,9]. Inflammation plays a key role in atherosclerosis, mediating all stages, from initiation through to progression [9,10].

Lower extremity chronic venous disease (LECVD) is characterized by symptoms and signs arising from morphological or functional abnormalities affecting the venous system [11]. One of its most common signs is varicose veins [11]. LECVD has a prevalence of 57% in men and 77% in women, while varicose veins are present in 25–33% of female adults and 10–40% of male adults [11,12].

In LECVD, there is a change in shear stress that causes leukocytes activation, adhesion, and migration through the endothelium [11]. The activated leukocytes produce cytokines, chemokines, growth factors, and proteases, resulting in an environment of persistent inflammation [13].

Inflammation is a common element in PAD and LECVD. This study aimed to assess whether the presence of PAD correlates with increased inflammatory cell infiltration. Our practical approach involved comparing the histological characteristics of skeletal muscle and adipose tissue—both perivascular adipose tissue (PVAT) and subcutaneous adipose tissue (SAT)—between patients diagnosed with PAD and those presenting with varicose veins. Patients with varicose veins were used in this study as controls due to the fact that they do not have macroscopic atherosclerotic disease. Through the exploration of these histological features, we aspire to contribute valuable insights into the intricate relationships between body composition, cardiovascular health, and the specific pathologies of PAD and LECVD.

## 2. Materials and Methods

### 2.1. Study Type and Inclusion/Exclusion Criteria

An observational, prospective study was conducted from January 2018 to July 2020 at a single institution in the Vascular Surgery and Internal Medicine departments. The study included consecutive patients (attending the vascular surgery consultation of the first author) with PAD, as suggested by clinical history and objective examination and confirmed with ABI and individuals with varicose veins without PAD who met the following criteria.

#### 2.1.1. Inclusion Criteria

Patients with PAD and with indication to surgical approach of common femoral artery.

Patients with varicose veins and in Class C2 from the CEAP classification, with indication for ligation of the saphenofemoral junction.

#### 2.1.2. Exclusion Criteria

- Bedridden individuals or subjects who refused to participate in the protocol.
- Those with diseases responsible for body composition changes or a pro-inflammatory state.
  - Recent diet change.
  - Active malignancy.
  - Auto-immune disease.
  - Active infection.
  - Chronic renal failure (GFR < 30 mL/min/1.73 m^2^).
  - Heart failure in the past 3 months

### 2.2. Ethical Considerations

Ethics approval for data collection and evaluation was obtained from the Ethics Committee of the Local Hospital (75/2017) and by the National Commission for Data Protection. The study was conducted according to the Declaration of Helsinki, as well as national and European guidelines for clinical research. All the participants signed an informed consent form.

### 2.3. Clinical Characteristics

Patients’ age, gender, and medication were registered. The following clinical data were collected by the main investigator and defined as stated in a previous publication: arterial hypertension, diabetes, dyslipidaemia, smoking habits, stroke/transient ischaemic attack, and medication (antiplatelet, statin-, and angiotensin-converting enzyme inhibitors—ACEIs) [14]. The following anthropometric data were assessed by a dedicated nurse: height, weight, BMI, WC, hip circumference (HC), and WHR. The methods used to determine these measures are described in a publication from our group [15].

### 2.4. C-Reactive Protein

A phlebotomy was performed in the morning after a 10–12 h fast. Blood samples were collected in appropriate vacutainer tubes, which were centrifuged within 5 min for 4000 cycle/min, and serum was separated. C-reactive protein (CRP) concentration was evaluated using routine procedures in the department of clinical chemistry.

### 2.5. Histologic Characteristics of Skeletal Muscle

In patients with surgical indication samples of sartorius skeletal muscle, SAT and PVAT (0.5 to 2 cm of the common femoral artery) were collected without the use of electrocautery. They were immediately preserved in formol and prepared for haematoxylin–eosin staining and immunohistochemical analysis.

#### 2.5.1. Haematoxylin–Eosin Analysis

The following characteristics of the skeletal muscle were registered: preservation of skeletal fibres; trauma (structure damage, such as the disruption of myofibrils or the sarcolemma); haemorrhage (accumulation of blood identified in the histology study); and the presence of inflammatory cells.

Examining the samples of adipose tissue (SAT and PVAT), we registered adipocyte preservation, the presence of adipocytes with imprecise limits, evidence of haemorrhage, and the presence of inflammatory cells

#### 2.5.2. Immunohistochemical Study

The samples of skeletal muscle and SAT were also subjected to immunohistochemical analysis for the detection of CD45+ leucocytes and CD163+ macrophages. Immunohistochemical staining was conducted with the HiDef Detection™ HRP Polymer System (Cell Marque™ Rocklin, CA, USA) to detect CD45+ leucocytes and CD163+ macrophages in samples of sartorius skeletal muscle. Primary antibodies were obtained from AbCam (CD45) and Cell Marque™ Rocklin, CA, USA (CD163). Antigen recovery was performed in 10 mM citrate buffer (pH 6.0) at 98 °C. Endogenous peroxidase activity was inactivated with 3% hydrogen peroxide in methanol. Primary antibodies were diluted at 1:500 (CD45) and 1:100 (CD163) dilutions and incubated on the sections for 120 min at room temperature. Staining was carried out using a liquid 3,3′-diaminobenzidine (DAB) substrate kit (Cell Marque™ Rocklin, CA, USA). The sections were counterstained with haematoxylin. Negative controls were analysed by omitting the primary antibodies. Placenta and tonsil sections were used as positive controls for CD45 and CD163 detection, respectively, as indicated by the manufacturers.

The amount of inflammatory cells (leucocytes and macrophages) was semi-quantitatively assessed using the following 0-to-4-grade scale: absent (0†), mild (†), moderate (††), severe (†††), and very severe (††††). The definitions of the grades stated above are as follows: mild (†): minimal focal infiltration of inflammatory cells; moderate (††): infiltration of inflammatory cells that occur at various points in the tissue sample without any change in the sample architecture; severe (†††): intense inflammatory infiltrate that may be associated with changes in tissue architecture or associated or not with necrosis and/or haemorrhage; very severe (††††): intense inflammatory infiltrate that is dispersed throughout the architecture of the tissue sample and that can sometimes hide the tissue pattern.

The samples were analysed by a pathologist with research experience who was blinded to the patients’ characteristics.

### 2.6. Statistical Analysis

The Shapiro–Wilk test was used to evaluate the distribution. Categorical variables are expressed as percentage, and smoking load are expressed as median and IQR. Categorical variables between the two groups were compared with a chi-square test; smoking load values were compared with the Mann–Whitney test, and CRP concentration was evaluated with Student’s t-test. Multiple linear regression was used to adjust for potential confounding variables. A *p*-value of less than 0.05 was considered significant. Statistical evaluation was performed using SPSS software, version 20.0 (SPSS, Inc., Chicago, IL, USA).

## 3. Results

### 3.1. Clinical Characteristics

A total of 55 patients, 44 with PAD (29 with chronic limb-threatening ischemia) and 11 with varicose veins, were enrolled in the study.

No differences were registered between the two groups regarding age, gender, and cardiovascular risk factors, except on smoking load (Table 1). There was a significantly higher smoking load in those with PAD than in patients with varicose veins [median = 30.00 pack years; IQR = 50.00; median = 0.00 pack years; IQR = 30.00; *p* = 0.002]. We observed that the patients with PAD also differed from the patients with varicose veins in terms of their use of antiplatelet and statin therapy (Table 1).

No differences were found between groups regarding WC (PAD:98.64 ± 10.19 cm; varicose veins patients: 100.56 ± 10.58 cm; *p* = 0.302) and WHR (PAD: median = 1.01 IQR = 0.1; varicose veins patients: median = 1.02 IQR = 0.09; *p* = 0.546). There appears to be a trend for patients with PAD to have a lower BMI compared to those with varicose veins, although this difference did not reach statistical significance (PAD: median = 26.06 Kg/m^2^ IQR = 5.21; varicose veins patients: median= 27.48 Kg/m^2^ IQR = 5.30; *p* = 0.057). On the other hand, patients with varicose veins had a higher HC when compared to those with PAD (PAD: median = 96 cm IQR = 9; varicose veins patients: median = 100 cm IQR = 12 *p* = 0.010). The multiple linear regression assessed the ability of smoking load, the presence of PAD, statins, and antiplatelet therapy to predict HC. The results revealed that smoking load (β = −0.201, t =−2.299, *p* = 0.23) and the presence of PAD (β = −0.174, t = −1.990, *p* = 0.049) were significant predictors of HC. The other variables had no impact.

### 3.2. Histologic Characteristics of Skeletal Muscle

Samples of sartorius skeletal muscle from 49 patients (41 with PAD and 8 with varicose veins) were analysed with haematoxylin–eosin staining. No differences were found in the preservation of skeletal muscle or the presence of muscle fibre trauma or haemorrhage (Table 2). Patients with PAD had a higher number of inflammatory cells

A total of 47 patients’ (39 with PAD and 8 with varicose veins) samples of sartorius muscle were analysed with immunohistology for CD45+ leucocytes, while 40 samples were analysed for CD163 macrophages (39 with PAD and 8 with varicose veins). No differences were found in this analysis (Table 3).

### 3.3. Histologic Characteristics of Adipose Tissue

**Figure 1:**
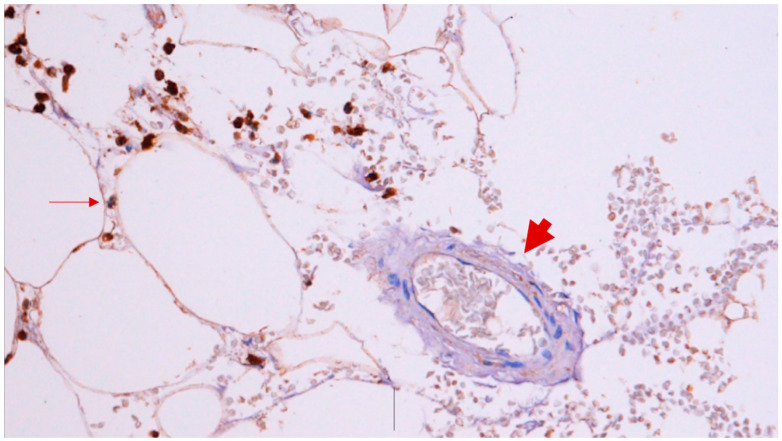
Immunohistology analysis of the SAT (CD45+ leucocytes) of a patient with PAD. The leucocytes are surrounding the adipocyte (thin arrow), and the vessel does not mark for leucocytes (biggest arrow).

Analysing the SAT samples of 44 patients with PAD and 11 patients with varicose veins, we found a higher quantity of CD45+ leucocytes in patients with PAD (Figure 1). No differences were found in the preservation of adipocytes or shape, haemorrhage, or number of inflammatory cells upon haematoxylin–eosin staining (Table 4 and Table 5).

The PVAT of patients with PAD (37), when compared to that of those with varicose veins (11), had a higher number of inflammatory cells. No other differences were identified (Table 6).

### 3.4. C-Reactive Protein

Patients with PAD had a higher serum level of CRP when compared to patients with varicose veins (18.07 ± 32.34 mg/L vs. 3.75 ± 3.00 mg/L *p* = 0.006).

## 4. Discussion

This is a preliminary study showing that patients with PAD have a higher number of inflammatory cells in the adipose tissue (PVAT, SAT) and skeletal muscle when compared to those with LECVD.

### 4.1. Anthropometric Measures, PAD, and LECVD

Analysing the anthropometric measures, we found that patients with PAD had a lower HC when compared to those with varicose veins. Our multiple linear regression analysis showed that both smoking load and the presence of PAD were associated with a lower HC, which is the best anthropometric measure to determine SAT [15].

A previous publication from our research team found that smoking load was negatively correlated with the area of SAT determined on CT scan in a cohort of vascular surgery patients, and these results could be explained by the inhibition of lipogenesis caused by nicotine inhalation [15,16].

However, as far as I know, there is no specific study stating that PAD patients have a lower HC when compared to those without PAD, but, despite controversy, PAD is associated with central obesity (higher WC and WHR) [7,17].

SAT is considered an anti-inflammatory fat depot that is protective against cardiometabolic disease, which helps to explain our results [18].

### 4.2. Skeletal Muscle, PAD, and LECVD

In this work we analysed the histological characteristics of the sartorius muscle to infer the characteristics of the central muscles. With this decision, we avoided samples of the skeletal muscle directly involved in the limb ischaemic process in PAD patients or the muscles involved in limb muscle pump dysfunction in LECVD patients [19]. We observed that patients with PAD had higher numbers of inflammatory cells. No additional differences were found in the other histological analysis. All previous published papers have analysed the histological characteristics of gastrocnemius muscle in patients with PAD [20,21,22,23]. They described a higher number of macrophages and collagen contents; changes in the characteristics of myofibre (atrophy, irregular shape, fibrosis, necrosis, and decrease in density); and variation in the number of capillaries (with some studies describing a higher number of capillaries and others describing a lower number of capillaries) [20,21,22,23]. As there is no other histologic study analysing the characteristics of the skeletal muscle (not directly affected by limb ischemia) in patients with and without PAD, we cannot make direct comparisons with our data. However, there is a previous (and unique) research work correlating the inflammation of the core skeletal muscle with atherosclerosis [24]. The authors of this study described that inflammation of psoas muscle determined with positron emission tomography was correlated with coronary artery disease [24]. This article also highlighted that inflammatory cells are the predominant cell populations in inflamed skeletal muscle [24]. Consequently, we can hypothesize that patients with PAD may have a higher inflammatory state in their core muscles when compared to patients without PAD. This inflammatory condition among PAD patients is supported by the increased serum concentration of CRP found in this group of patients in comparison to those with varicose veins. In fact, systemic inflammation is considered an important pathophysiological mechanism in PAD and likely to contribute to skeletal muscle wasting [21,25].

### 4.3. Adipose Tissue, PAD, and LECVD

In patients with PAD, we found a higher number of CD45+ leucocytes in the immunohistochemical analysis of SAT and higher number of inflammatory cells in PVAT analysed via haematoxylin–eosin staining compared to those with varicose veins. These data emphasize that adipose tissue in PAD patients is characterized by the presence of inflammatory cells, with this group of patients being more frequently medicated with statins. Statins are able to decrease the proportion of pro-inflammatory macrophages in visceral adipose tissue [26].

As far we know, just one study has analysed the inflammatory cells in the adipose tissue of PAD patients [27]. In this study, the authors compared the number of macrophages in the adipose tissue (visceral, PVAT, and SAT) of 24 PAD patients with that of 35 living kidney donors [27]. The authors concluded that there were no differences in visceral and PVAT, but PAD patients had higher numbers of macrophages in SAT [27]. We did not register differences in the quantity of CD163+ macrophages. However, this research work cannot be directly compared with ours. The authors only analysed CD14+ macrophages and did not clearly state in which anatomic region the SAT was collected [27]. In our study, we collected adipose tissue at the femoral region, and the tissue collected from this region could be different from that at the abdominal wall or other anatomical region.

We also observed a higher number of inflammatory cells in PVAT compared to patients with varicose veins. Similarly, Poledne et al. found a higher macrophage content in adipose tissue surrounding the coronary artery in patients with ischaemic heart disease but not in patients transplanted for cardiomyopathy [28]. Other works proved that PVAT surrounding atherosclerotic arteries is rich in inflammatory markers [29]. In peri-plaque coronary adipose tissue, the density of CD68+ macrophages and CD20+ B lymphocytes increased with the size of the plaque, and coronary PVAT surrounding unstable atherosclerotic plaques exhibited greater CD68+ macrophages than surrounding stable lesions [29]. In patients with diabetes, carotid PVAT surrounding the atheromatous plaques showed an increase in the mRNA levels of tumour necrosis factor-α (TNF-α), monocyte chemoattractant protein-1 (MCP-1), and interleukin-6 (IL-6) [29].

**Figure 2:**
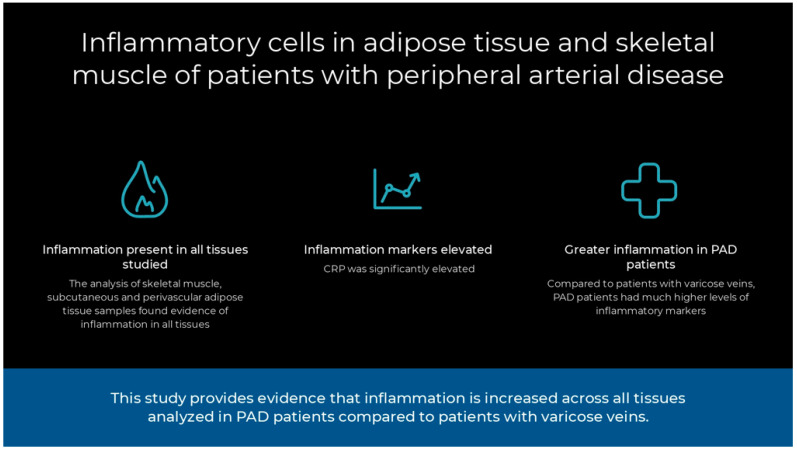
Picture summarizing the main results of this study.

Our results emphasize that adipose tissue in PAD patients is characterized by the presence of inflammatory cells, which is in accordance with the literature, when compared to patients with chronic venous disease. Čejková et al., analysing samples of adipose tissue, found that patients with PAD have a higher expression of inflammatory genes in their adipose tissue (visceral, SAT, PVAT) [27] (Figure 2).

### 4.4. Limitations and Aims for a Future Investigation

This is a preliminary study that needs further research to validate its results. We analysed the histology of the two main tissues, skeletal muscle and adipose tissue, in patients with PAD. Few research works have compared histological characteristics between patients with and without PAD, and no previous study has compared between the core muscles of these two groups of patients. In spite of this fact, our results are in line with the literature and seem to show that inflammation is a characteristic of PAD that extends beyond its serum levels and is present in the tissues of PAD patients. Consequently, a decrease in inflammation should be a priority in this population. Statins, angiotensin-converting enzyme inhibitors, and angiotensin receptor blockers are examples of therapies that decrease inflammation in adipose tissue, at least in visceral fat [15].

This research work has several limitations: it was conducted at a single centre; the control group was not a truly healthy control population; and we did not evaluate the immunohistochemical characteristics of PVAT due to budget constraints. The control group was populated by individuals without PAD but with varicose veins, a pathology that affects body composition, which may have potentially confounded the results, thereby limiting the generalizability of the findings. Another limitation is the presence of selection bias, as some potential participants refused to take part, and a significant number of bedridden individuals were excluded. Additionally, more than 50% of PAD patients had chronic limb-threatening ischemia, a clinical state associated with serum markers of inflammation [14]. Patients with chronic limb-threatening ischemia may also have wound infection (although we tried to exclude such cases), which could be a confounder. Due to the small sample size and measurement errors, this study has a risk of type II statistical errors. The small sample also limited our ability to use multivariate testing and our capacity to identify true relationships between the variables, decreasing the generalization of the results. We recognize that the sample size not only contributes to the risk of type II statistical errors but also constrains our ability to conduct robust multivariate testing.

It is an aim of our team to carry on with this work. We intend to perform an immunohistochemical analysis of PVAT and to correlate this analysis with the severity of atherosclerosis. We also would like to better characterize the subpopulation of macrophages in type M1 and type M2. We will study the presence of myokines and adipokines in both adipose tissue and skeletal muscle samples. Additionally, we intend to examine histologic inflammation in relation to PAD patient outcomes and explore the correlation between the histologic characteristics of the skeletal muscle with its macroscopic features.

## 5. Conclusions

Patients with PAD have a higher number of inflammatory cells in the histology of SAT, PVAT, and skeletal muscle compared to those with varicose veins, emphasizing the role of inflammation in their tissues. The correlation with systemic inflammation would be an interesting point to investigate.

## Figures and Tables

**Table 1:** Demographic characteristics, atherosclerotic risk factors, and medication of PAD patients and those with varicose veins analysed in this study. * *p* < 0.05 is considered significant (TIA—transient ischaemic attack; ACEIs—angiotensin-converting enzyme inhibitors).

	PAD (n = 44)	Patients with Varicose Veins (n = 11)	*p*-Value
Male (n; %)	34; 77.27	9; 81.81	0.497
Age (years-old)	66.98 ± 9.92	65.09 ± 9.55	0.575
Hypertension (n; %)	26; 59.09	37; 63.64	0.916
Dyslipidaemia (n; %)	28; 63.64	5; 45.45	0.196
Smoker/ex-smoker (n; %)	27; 61.36	8; 72.73	0.387
Diabetes (n; %)	17; 38.64	4; 36.36	0.804
Coronary artery disease (n; %)	7; 15.91	1; 9.91	0.532
Stroke/TIA (n;%)	3; 6.82	1; 9.91	0.468
Antiplatelet (n;%)	35; 79.54	5; 45.45	0.000 *
Statin (n;%)	38; 86.36	7; 63.64	0.004 *
ACEI (n;%)	14; 31.82	3; 27.27	0.981
Beta-blockers (n;%)	11; 25.00	3; 27.27	0.154

**Table 2:** Histologic characteristics of skeletal muscle found following haematoxylin–eosin staining of patients with PAD and varicose veins analysed in this study. * *p* < 0.05 is considered significant.

	PAD (n = 41)	Patients with Varicose Veins (n = 8)	X^2^	df	*p*-Value	Phi
Well-preserved skeletal fibres	26; 63.41	3; 37.50	1.861	1	0.173	0.195
Trauma (n; %)	4; 9.76	2; 25.00	1.448	1	0.229	−0.172
Haemorrhage (n; %)	6; 14.63	0; 0	1.334	1	0.248	0.165
Inflammatory cells	18; 43.90	0; 0	5.552	1	0.018 *	0.337

**Table 3:** Immunohistochemical characteristics of skeletal muscle of patients with PAD and varicose veins.

	PAD (n = 39)	Patients with Varicose Veins (n = 8)	X^2^	df	*p*-Value	Phi
CD 45+ mild or absent (0;†) (n/%)	19; 48.72	5; 62.50	0.505	1	0.477	−0.104
CD 45+ > moderate (††; †††; ††††) (n/%)	20; 51.28	3; 37.50				
CD 163+ mild or absent (0;†) (n/%)	31; 77.50	7; 87.50	0.544	1	0.461	−0.105
CD 163+ > moderate (††; †††; ††††) (n/%)	9; 22.50	1; 12.50				

**Table 4:** Histologic characteristics of SAT found after haematoxylin–eosin staining of patients with PAD and patients with varicose veins.

	PAD (n = 44)	Patients with Varicose Veins (n = 11)	X^2^	df	*p*-Value	Phi
Well-preserved adipocytes (n; %)	11; 25.00	3; 27.27	0.024	1	0.877	0.195
Adipocytes imprecise limits (n; %)	27; 61.36	5; 45.45	0.915	1	0.339	−0.172
Haemorrhage (n; %)	6; 13.64	1; 9.09	0.164	1	0.686	0.165
Inflammatory cells	6; 13.64	2; 18.18	0.146	1	0.702	0.337

**Table 5:** Immunohistochemical characteristics of SAT of patients with PAD and varicose. * *p* < 0.05 is considered significant.

	PAD (n = 44)	Patients with Varicose Veins (n = 11)	X^2^	df	*p*-Value	Phi
CD 45+ mild or absent (0;†) (n/%)	12; 27.27	8; 72.73	7.857	1	0.005 *	−0.378
CD 45+ > moderate (††; †††; ††††) (n/%)	32; 72.73	3; 27.27				
	**(n = 42)**	**(n = 11)**				
CD 163+ mild or absent (0;†) (n/%)	21; 50.00	7; 63.64	0.859	1	0.345	−0.127
CD 163+ > moderate (††; †††; ††††) (n/%)	21; 50.00	4; 36.36				

**Table 6:** Histologic characteristics of PVAT found following haematoxylin–eosin staining of patients with PAD and varicose veins analysed in this study. * *p* < 0.05 is considered significant.

	PAD (n = 37)	Patients with Varicose Veins (n = 11)	X^2^	df	*p*-Value	Phi
Well-preserved adipocytes (n; %)	14; 37.84	5; 45.45	0.206	1	0.650	−0.065
Adipocytes imprecise limits (n; %)	17; 45.95	6; 54.54	0.251	1	0.616	−0.072
Haemorrhage (n; %)	2; 5.41	0; 0	0.620	1	0.431	0.114
Inflammatory cells	16; 43.24	0; 0	7.135	1	0.008 *	0.386

## Data Availability

The data that support the findings of this study are available from the corresponding author (J.F.).
